# Crystal structure, Hirshfeld surface analysis, inter­action energy and DFT studies of (2*Z*)-2-(2,4-di­chloro­benzyl­idene)-4-nonyl-3,4-di­hydro-2*H*-1,4-benzo­thia­zin-3-one

**DOI:** 10.1107/S2056989020001036

**Published:** 2020-01-31

**Authors:** Brahim Hni, Nada Kheira Sebbar, Tuncer Hökelek, Achour Redouane, Joel T. Mague, Noureddine Hamou Ahabchane, El Mokhtar Essassi

**Affiliations:** a Laboratoire de Chimie Organique Hétérocyclique URAC 21, Pôle de Compétence Pharmacochimie, Av. Ibn Battouta, BP 1014, Faculté des Sciences, Université Mohammed V, Rabat, Morocco; b Laboratoire de Chimie Appliquée et Environnement, Equipe de Chimie Bioorganique Appliquée, Faculté des Sciences, Université Ibn Zohr, Agadir, Morocco; c Department of Physics, Hacettepe University, 06800 Beytepe, Ankara, Turkey; d Department of Chemistry, Tulane University, New Orleans, LA 70118, USA

**Keywords:** crystal structure, 1,4-benzo­thia­zin-3-one, di­hydro­thia­zine, hydrogen bond, π-stacking, Hirshfeld surface

## Abstract

The title compound, C_24_H_27_Cl_2_NOS, contains 1,4-benzo­thia­zine and 2,4-di­chloro­phenyl­methyl­idene units in which the di­hydro­thia­zine ring adopts a screw-boat conformation. In the crystal, inter­molecular C—H_Bnz_⋯O_Thz_ (Bnz = benzene and Thz = thia­zine) hydrogen bonds form chains of mol­ecules extending along the *a*-axis direction, which are connected to their inversion-related counterparts by C—H_Bnz_⋯Cl_Dchlphy_ (Dchlphy = 2,4-di­chloro­phen­yl) hydrogen bonds and C—H_Dchlphy_⋯π (ring) inter­actions. These double chains are further linked by C—H_Dchlphy_⋯O_Thz_ hydrogen bonds, forming stepped layers approximately parallel to (012). The Hirshfeld surface analysis of the crystal structure indicates that the most important contributions for the crystal packing are from H⋯H (44.7%), C⋯H/H⋯C (23.7%), Cl⋯H/H⋯Cl (18.9%), O⋯H/H⋯O (5.0%) and S⋯H/H⋯S (4.8%) inter­actions. Hydrogen-bonding and van der Waals inter­actions are the dominant inter­actions in the crystal packing. Computational chemistry indicates that in the crystal, C—H_Dchlphy_⋯O_Thz_, C—H_Bnz_⋯O_Thz_ and C—H_Bnz_⋯Cl_Dchlphy_ hydrogen-bond energies are 134.3, 71.2 and 34.4 kJ mol^−1^, respectively. Density functional theory (DFT) optimized structures at the B3LYP/6–311 G(d,p) level are compared with the experimentally determined mol­ecular structure in the solid state. The HOMO–LUMO behaviour was elucidated to determine the energy gap. The two carbon atoms at the end of the nonyl chain are disordered in a 0.562 (4)/0.438 (4) ratio.

## Abstract 2

The title compound contains 1,4-benzo­thia­zine and 2,4-di­chloro­phenyl­methyl­idene units in which the di­hydro­thia­zine ring adopts a screw-boat conformation. In the crystal, inter­molecular C—H_Bnz_⋯O_Thz_ (Bnz = benzene and Thz = thia­zine) hydrogen bonds form chains of mol­ecules extending along the *a*-axis direction which are connected to their inversion-related counterparts by C—H_Bnz_⋯Cl_Dchlphy_ (Dchlphy = 2,4-di­chloro­phen­yl) hydrogen bonds and C—H_Dchlphy_⋯π (ring) inter­actions. These double chains are further linked by C—H_Dchlphy_⋯O_Thz_ hydrogen bonds to form stepped layers approximately parallel to (012).

## Chemical context   

A number of sulfur- and nitro­gen-containing heterocyclic compounds have been well studied. These mol­ecules exhibit a wide range of biological applications, indicating that the 1,4-benzo­thia­zine moiety is a potentially useful template in medicinal chemistry research with therapeutic applications in the anti­microbial (Armenise *et al.*, 2012 ▸, Sabatini *et al.*, 2008 ▸), anti-viral (Malagu *et al.*, 1998 ▸), anti-oxidant (Zia-ur-Rehman *et al.* 2009 ▸), anti-inflammatory (Trapani *et al.*, 1985 ▸; Gowda *et al.*, 2011 ▸) anti­pyretic (Warren *et al.*, 1987 ▸), and anti-cancer (Gupta *et al.*, 1991 ▸; Gupta *et al.*, 1985 ▸) areas as well as being precursors for the synthesis of new compounds (Sebbar *et al.*, 2015*a* ▸; Vidal *et al.*, 2006 ▸) possessing anti-diabetic (Tawada *et al.*, 1990 ▸) and anti-corrosion activities (Ellouz *et al.*, 2016*a* ▸,*b* ▸; Sebbar *et al.*, 2016*a* ▸) and biological properties (Hni *et al.*, 2019*a* ▸; Ellouz *et al.*, 2017*a* ▸,*b* ▸, 2018 ▸; Sebbar *et al.*, 2019*a* ▸,*b* ▸). As a continuation of our research into the development of new 1,4-benzo­thia­zine derivatives with potential pharmacological applications, we have studied the reaction of 1-bromo­nonane with (*Z*)-2-(2,4-di­chloro­benzyl­idene)-2*H*-1,4-benzo­thia­zin-3(4*H*)-one under phase-transfer catalysis conditions using tetra-*n*-butyl­ammonium bromide (TBAB) as catalyst and potassium carbonate as base (Hni *et al.*, 2019*b* ▸; Sebbar *et al.*, 2019) to give the title compound, (I), in good yield. We report here its crystalline and mol­ecular structures as well as the Hirshfeld surface analysis and the density functional theory (DFT) computational calculations.
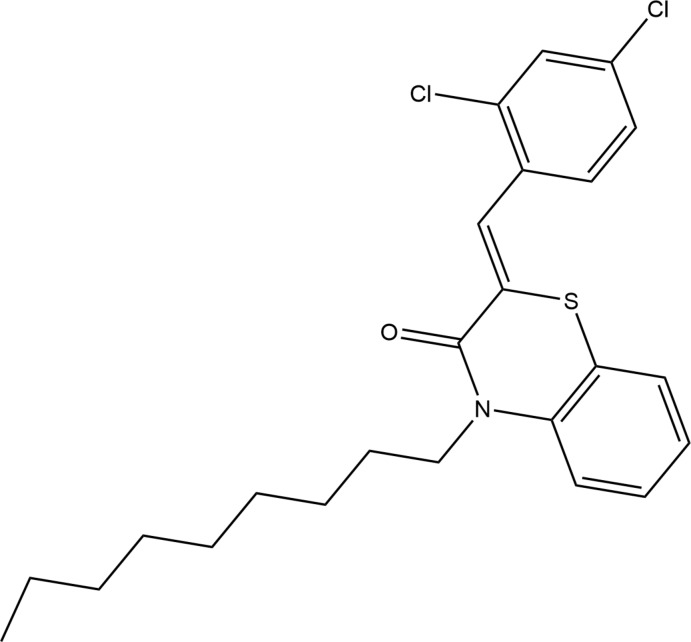


## Structural commentary   

**Figure 1:**
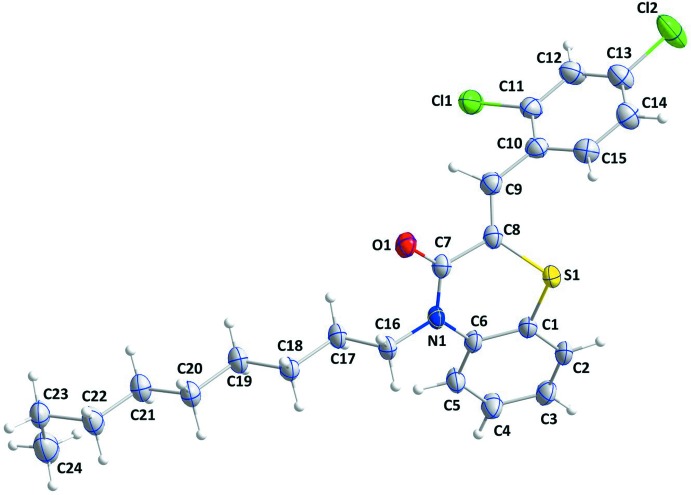
The mol­ecular structure of the title compound with the atom-numbering scheme. Displacement ellipsoids are drawn at the 50% probability level.

The title compound contains 1,4-benzo­thia­zine and 2,4-di­chloro­phenyl­methyl­idene units (Fig. 1 ▸), in which the di­hydro­thia­zine ring, *B* (S1/N1/C1/C6–C8), adopts a screw-boat conformation with puckering parameters *Q*_T_ = 0.5581 (16) Å, θ = 69.76 (18)° and φ = 334.3 (2)°. The planar rings, *A* (C1–C6) and *C* (C10–C15) are oriented at a dihedral angle of 88.45 (7)°. Atoms Cl1, Cl2 and C9 are almost co-planar with ring *C* being displaced by 0.0247 (6), −0.0732 (9) and −0.0274 (2) Å, respectively.

## Supra­molecular features   

**Figure 2:**
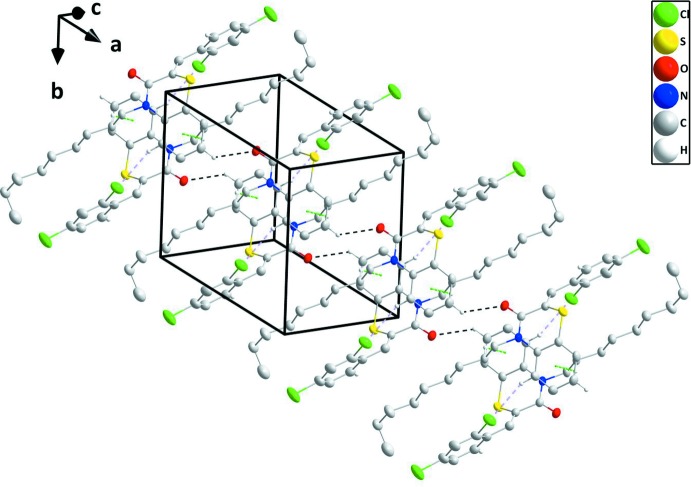
A perspective view of one double chain. The inter­molecular C—H_Bnz_⋯O_Thz_ and C—H_Bnz_⋯Cl_Dchlphy_ (Bnz = benzene,Thz = thia­zine and Dchlphy = 2,4-di­chloro­phen­yl) hydrogen bonds are shown, respectively, as black and light purple dashed lines while the C—H_Dchlphy_⋯π (ring) inter­actions are shown as green dashed lines.

In the crystal, C—H_Bnz_⋯O_Thz_ (Bnz = benzene and Thz = thia­zine) hydrogen bonds link the mol­ecules, forming chains extending along the *a*-axis direction, which are connected to their inversion-related counterparts by C—H_Bnz_⋯Cl_Dchlphy_ (Dchlphy = 2,4-di­chloro­phen­yl) hydrogen bonds and C—H_Dchlphy_⋯π (ring) inter­actions (Table 1 ▸ and Fig. 2 ▸). These double chains are further linked by C—H_Dchlphy_⋯O_Thz_ hydrogen bonds to form stepped layers approximately parallel to (012) (Table 1 ▸ and Figs. 2 ▸ and 3 ▸).

## Hirshfeld surface analysis   

**Figure 4:**
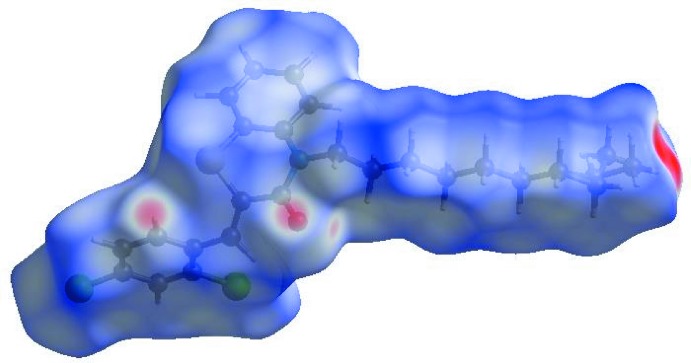
View of the three-dimensional Hirshfeld surface of the title compound plotted over *d*_norm_ in the range −0.6343 to 1.4076 a.u.

**Figure 5:**
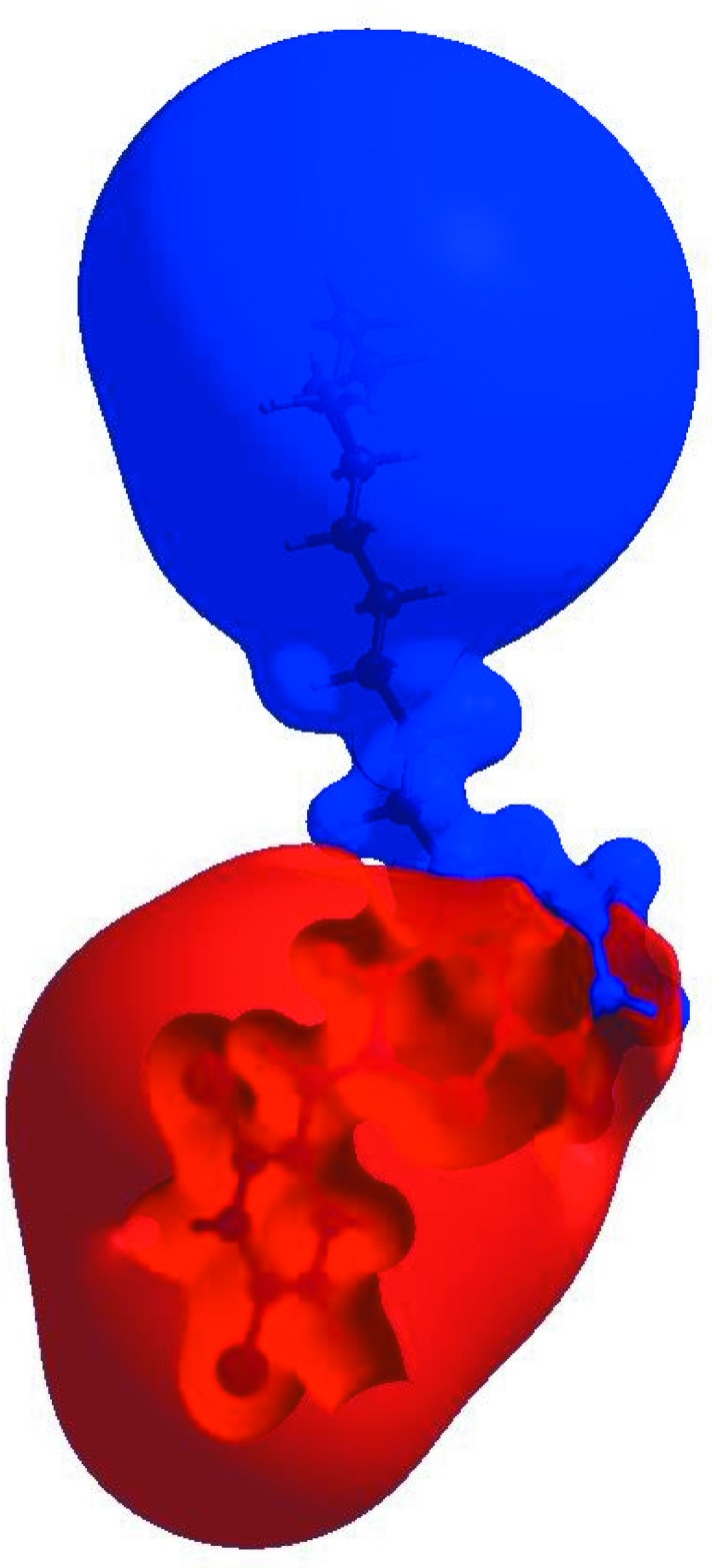
View of the three-dimensional Hirshfeld surface of the title compound plotted over electrostatic potential energy in the range −0.0500 to 0.0500 a.u. using the STO-3 G basis set at the Hartree–Fock level of theory. Hydrogen-bond donors and acceptors are shown as blue and red regions around the atoms corresponding to positive and negative potentials, respectively.

**Figure 6:**
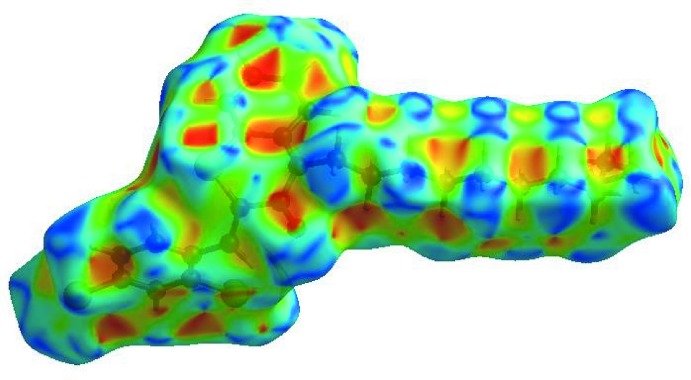
Hirshfeld surface of the title compound plotted over shape-index.

**Figure 7:**
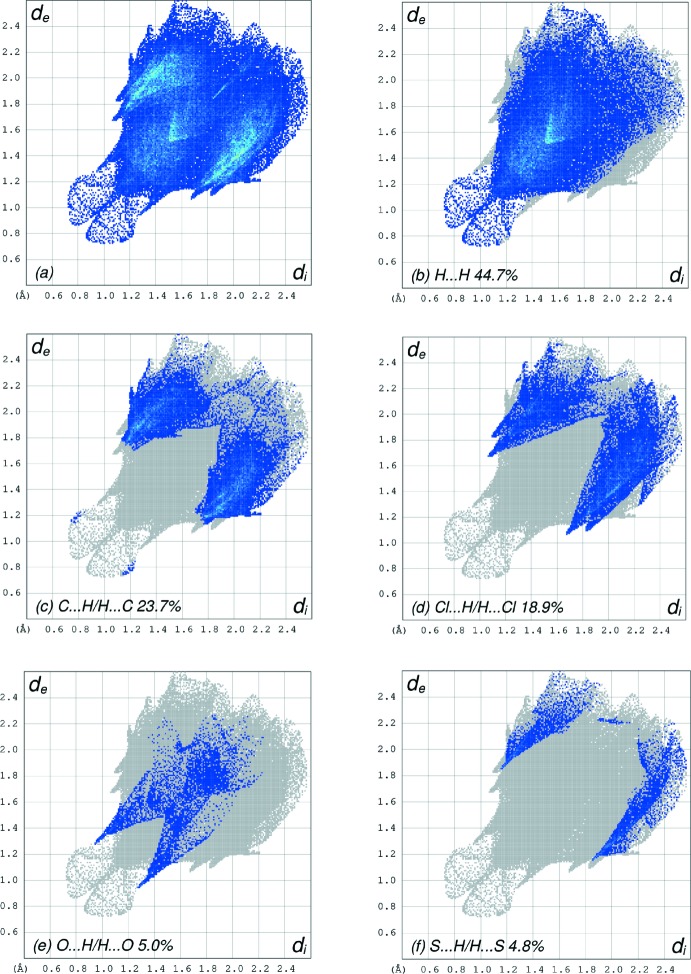
The full two-dimensional fingerprint plots for the title compound, showing (*a*) all inter­actions, and those delineated into (*b*) H⋯H, (*c*) C⋯H/H⋯C, (*d*) Cl⋯H/H⋯Cl, (*e*) O⋯H/H ⋯ O and (*f*) S⋯H/H⋯S contacts. The *d*_i_ and *d*_e_ values are the closest inter­nal and external distances (in Å) from given points on the Hirshfeld surface contacts.

In order to visualize the inter­molecular inter­actions in the crystal of the title compound, a Hirshfeld surface (HS) analysis (Hirshfeld, 1977 ▸; Spackman & Jayatilaka, 2009 ▸) was carried out by using *Crystal Explorer 17.5* (Turner *et al.*, 2017 ▸). In the HS plotted over *d*_norm_ (Fig. 4 ▸), the white surface indicates contacts with distances equal to the sum of van der Waals radii, and the red and blue colours indicate distances shorter (in close contact) or longer (distinct contact) than the van der Waals radii (Venkatesan *et al.*, 2016 ▸). The bright-red spots appearing near O1 and hydrogen atom H15 indicate their roles as the respective donors and/or acceptors; they also appear as blue and red regions corresponding to positive and negative potentials on the HS mapped over electrostatic potential (Spackman *et al.*, 2008 ▸; Jayatilaka *et al.*, 2005 ▸) as shown in Fig. 5 ▸. The blue regions indicate positive electrostatic potential (hydrogen-bond donors), while the red regions indicate negative electrostatic potential (hydrogen-bond acceptors). The shape-index of the HS is a tool to visualize π–π stacking by the presence of adjacent red and blue triangles; if there are no adjacent red and/or blue triangles, then there are no π–π inter­actions. Fig. 6 ▸ clearly suggests that there are no π–π inter­actions in (I). The overall two-dimensional fingerprint plot, Fig. 7 ▸ *a*, and those delineated into H⋯H, C⋯H/H⋯C, Cl⋯H/H ⋯ Cl, O⋯H/H⋯O and S⋯H/H⋯S contacts (McKinnon *et al.*, 2007 ▸) are illustrated in Fig. 7 ▸ *b*–*f*, respectively, together with their relative contributions to the Hirshfeld surface. The most important inter­action is H⋯H (Table 2 ▸), contributing 44.7% to the overall crystal packing, which is reflected in Fig. 7 ▸ *b* as widely scattered points of high density due to the large hydrogen content of the mol­ecule with the tip at *d*_e_ = *d*_i_ = 1.09 Å. The presence of C—H⋯π inter­actions is indicated by the fringed pairs of characteristic wings in the fingerprint plot delineated into C⋯H/H⋯C contacts (Fig. 7 ▸ *c*, 23.7% contribution to the HS). The two pairs of wings in the fingerprint plot delineated into Cl⋯Hl/H⋯Cl contacts (Fig. 7 ▸ *d*, 18.9% contribution) have an unsymmetrical distribution of points due to a third wing, with the edges at *d*_e_ + *d*_i_ = 2.74 Å (for the long wing), *d*_e_ + *d*_i_ = 2.92 Å (for the short wing) and *d*_e_ + *d*_i_ = 3.53 Å (for the unsymmetrical third wing). The pair of wings in the fingerprint plot delineated into O⋯H/H⋯O contacts (Fig. 7 ▸ *e*, 5.0% contribution) has a pair of spikes with the tips at *d*_e_ + *d*_i_ = 2.22 Å. Finally, the wings in the fingerprint plot delineated into S⋯H/H⋯S contacts (Fig. 7 ▸ *f*, 4.8% contribution) have the tips at *d*_e_ + *d*_i_ = 2.99 Å.

**Figure 8:**
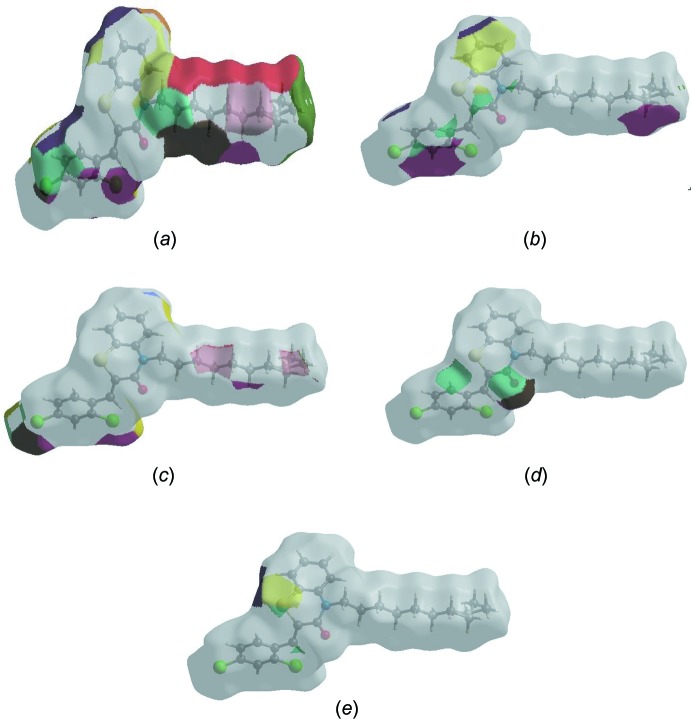
The Hirshfeld surface representations with the function *d*_norm_ plotted onto the surface for (*a*) H⋯H, (*b*) C⋯H/H⋯C, (*c*) Cl ⋯ H/H⋯Cl, (*d*) O⋯H/H⋯O and (*f*) S⋯H/H⋯S inter­actions.

The Hirshfeld surface representations with the function *d*_norm_ plotted onto the surface are shown for the H⋯H, C⋯H/H⋯C, Cl⋯H/H⋯Cl, O⋯H/H⋯O and S⋯H/H⋯S inter­actions in Fig. 8 ▸ *a*–*e*, respectively.

The Hirshfeld surface analysis confirms the importance of H-atom contacts in establishing the packing. The large number of H⋯H, C⋯H/H⋯C, Cl⋯H/H⋯Cl and O⋯H/H⋯O inter­actions suggest that van der Waals inter­actions and hydrogen bonding play the major roles in the crystal packing (Hathwar *et al.*, 2015 ▸).

## Inter­action energy calculations   

The inter­molecular inter­action energies were calculated using the CE–B3LYP/6–31G(d,p) energy model available in *Crystal Explorer 17.5* (Turner *et al.*, 2017 ▸), where a cluster of mol­ecules is generated by applying crystallographic symmetry operations with respect to a selected central mol­ecule within the default radius of 3.8 Å (Turner *et al.*, 2014 ▸). The total inter­molecular energy (*E*_tot_) is the sum of electrostatic (*E*_ele_), polarization (*E*_pol_), dispersion (*E*_dis_) and exchange-repulsion (*E*_rep_) energies (Turner *et al.*, 2015 ▸) with scale factors of 1.057, 0.740, 0.871 and 0.618, respectively (Mackenzie *et al.*, 2017 ▸). Hydrogen-bonding inter­action energies (in kJ mol^−1^) were calculated to be −53.7 (*E*_ele_), −13.6 (*E*_pol_), −161.9 (*E*_dis_), 119.0 (*E*_rep_) and −134.3 (*E*_tot_) for C—H_Dchlphy_⋯O_Thz_, 25.6 (*E*_ele_), −5.7 (*E*_pol_), −62.1 (*E*_dis_), 23.1 (*E*_rep_) and −71.2 (*E*_tot_) [or C—H_Bnz_⋯O_Thz_ and −16.0 (*E*_ele_), −8.3 (*E*_pol_), −43.0 (*E*_dis_), 42.2 (*E*_rep_) and −34.4 (*E_t_*_ot_) for C—H_Bnz_⋯Cl_Dchlphy_ (Bnz = benzene, Thz = thia­zine and Dchlphy = 2,4-di­chloro­phen­yl).

## DFT calculations   

**Figure 9:**
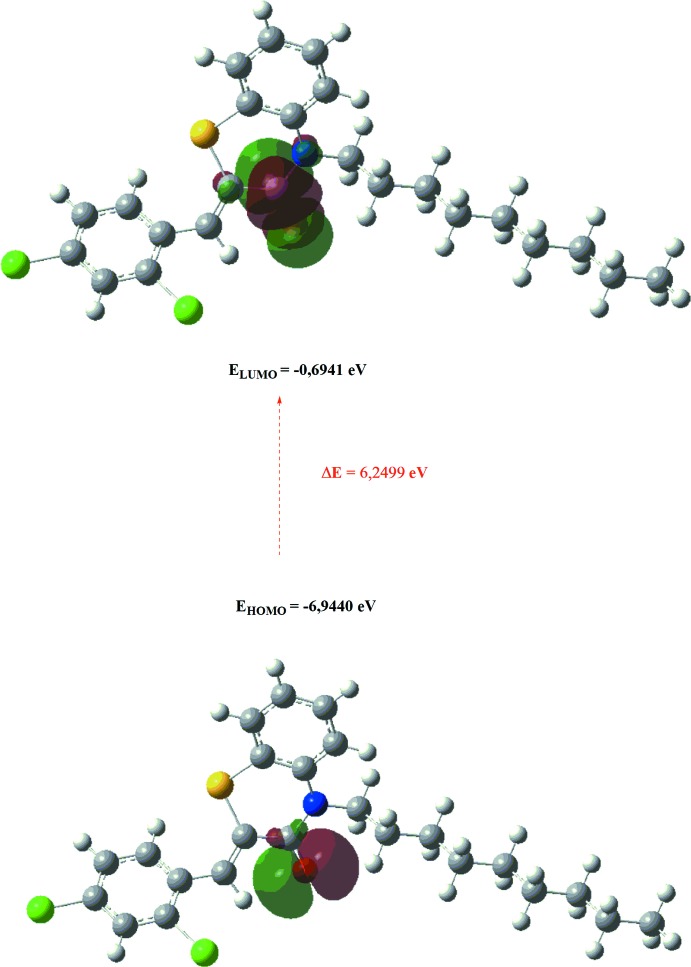
The energy band gap of the title compound.

The optimized structure of the title compound, (I), in the gas phase was generated theoretically *via* density functional theory (DFT) using the standard B3LYP functional and 6–311G(d,p) basis-set calculations as implemented in *GAUSSIAN 09* (Frisch *et al.*, 2009 ▸). The theoretical and experimental results are in good agreement (Table 3 ▸). The highest-occupied mol­ecular orbital (HOMO), acting as an electron donor, and the lowest-unoccupied mol­ecular orbital (LUMO), acting as an electron acceptor, are very important parameters for quantum chemistry. When the energy gap is small, the mol­ecule is highly polarizable and has high chemical reactivity. The DFT calculations provide some important information on the reactivity and site selectivity of the mol­ecular framework. *E*_HOMO_ and *E*_LUMO_ clarify the inevitable charge-exchange collaboration inside the studied material, and together with the electronegativity (χ), hardness (η), potential (μ), electrophilicity (ω) and softness (*σ*) are recorded in Table 4 ▸. The significance of η and *σ* is to evaluate both the reactivity and stability. The electron transition from the HOMO to the LUMO energy level is shown in Fig. 9 ▸. The HOMO and LUMO are localized in the plane extending from the whole (2*Z*)-2-[(2,4-di­chloro­phen­yl)methyl­idene]-4-nonyl-3,4-di­hydro-2*H*-1,4-benzo­thia­zin-3-one ring.

## Database survey   

A search in the Cambridge Structural Database (Groom *et al.*, 2016 ▸; updated to October 2019), for compounds containing the fragment **II** gave 14 hits.
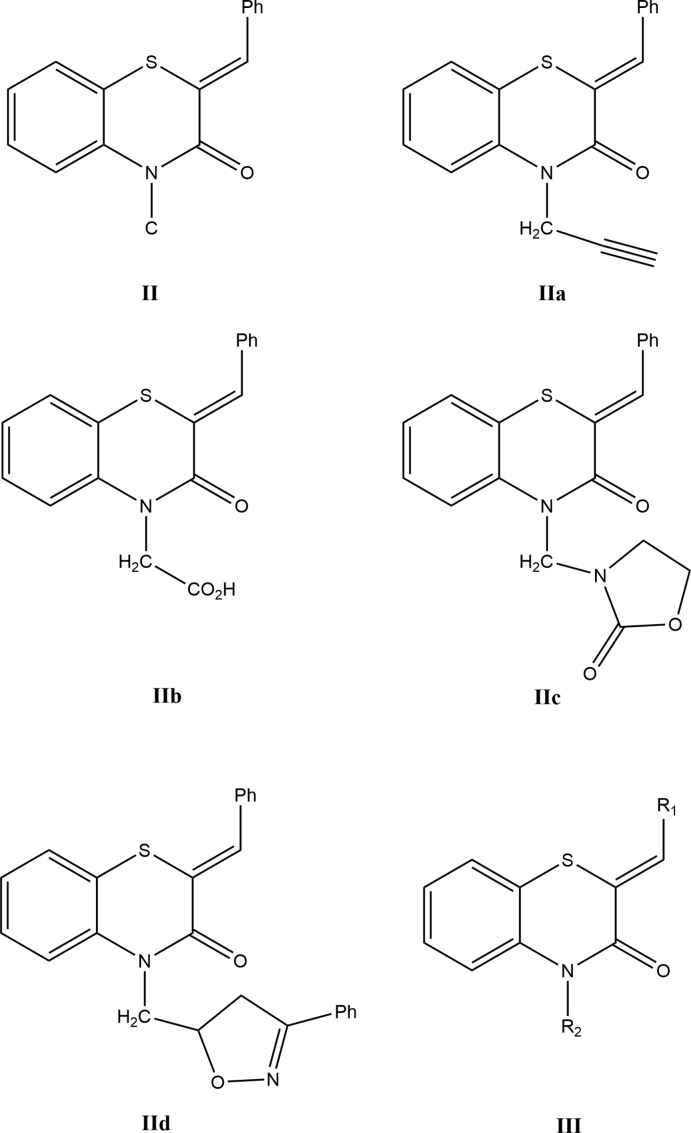


The largest set contains **IIa** (COGRUN; Sebbar *et al.*, 2014*a* ▸), **IIb** (APAJUY; Sebbar *et al.*, 2016*c* ▸), **IIc** (EVIYIT; Sebbar *et al.*, 2016*b* ▸) and **IId** (WUFGIP; Sebbar *et al.*, 2015*b* ▸). Additional examples are **III**: *R*_1_ = 4-FC_6_H_4_ and *R*_2_ = CH_2_C≡CH (WOCFUS; Hni *et al.*, 2019*a* ▸), *R*_1_ = 4-ClC_6_H_4_ and *R*_2_ = CH_2_Ph (OMEGEU; Ellouz *et al.*, 2016*c* ▸) and *R*_1_ = 2-ClC_6_H_4_, *R*_2_ = CH_2_C≡CH (SAVTUH; Sebbar *et al.*, 2017 ▸). In all these compounds, the configuration about the benzyl­idene group: C=CHC_6_H_5_ bond is *Z*, and in the majority of these, the heterocyclic ring is quite non-planar with the dihedral angle between the plane defined by the benzene ring plus the nitro­gen and sulfur atoms and that defined by nitro­gen and sulfur and the other two carbon atoms separating them having approximate values of 36° (WUFGIP), 29° (APAJUY), 28° (SAVTUH), 26° (WOCFUS) and 25° (COGRUN). By contrast, in both EVIYIT and OMEGEU, the benzo­thia­zine unit is nearly planar with the corresponding dihedral angle being about 4°.

## Synthesis and crystallization   

To a solution of (*Z*)-2-(2,4-di­chloro­benzyl­idene)-2*H*-1,4-benzo­thia­zin-3(4*H*)-one (1.5 mmol), potassium carbonate (2.7 mmol) and tetra-*n*-butyl ammonium bromide (0.14 mmol) in DMF (20 mL) was added 1-bromo­nonane (2.6 mmol). Stirring was continued at room temperature for 24 h. The mixture was filtered and the solvent removed. The residue obtained was washed with water. The organic compound was chromatographed on a column of silica gel with ethyl acetate–hexane (9/1) as eluent. Colourless crystals were isolated when the solvent was allowed to evaporate (yield = 79%).

## Refinement   

Crystal data, data collection and structure refinement details are summarized in Table 5 ▸. The two carbon atoms at the end of the nonyl chain, C23 and C24, are disordered in a 0.562 (4)/0.438 (4) ratio. These were refined with restraints that the two components have comparable geometries. The H atoms on these carbons as well as those on C22 were included as riding contributions in idealized positions (C—H = 0.99 Å with *U*_iso_(H) = 1.5*U*_eq_(C).

## Supplementary Material

Crystal structure: contains datablock(s) I, global. DOI: 10.1107/S2056989020001036/lh5943sup1.cif

Structure factors: contains datablock(s) I. DOI: 10.1107/S2056989020001036/lh5943Isup2.hkl

Supporting information file. DOI: 10.1107/S2056989020001036/lh5943Isup3.cdx

Supporting information file. DOI: 10.1107/S2056989020001036/lh5943Isup4.cml

CCDC reference: 1980073

Additional supporting information:  crystallographic information; 3D view; checkCIF report

## Figures and Tables

**Figure 3:**
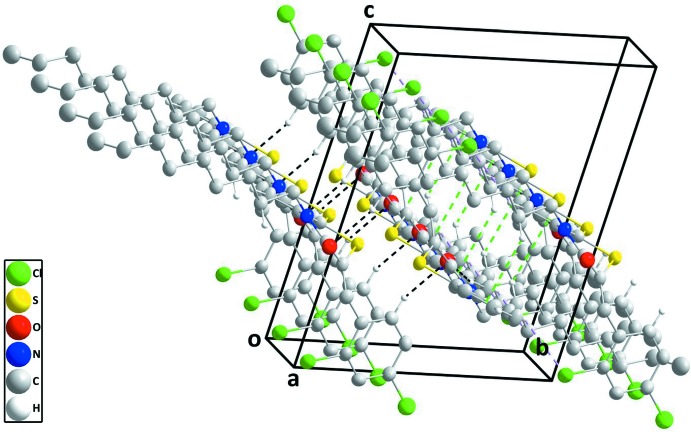
Perspective view of one double chain and half of a second showing the C—H_Dchlphy_⋯O_Thz_ (Dchlphy = 2,4-di­chloro­phenyl and Thz = thia­zine) hydrogen bond connecting them. Inter­molecular inter­actions depicted as in Fig. 2 ▸.

**Table 1:** Hydrogen-bond geometry (Å, °) *Cg*1 is the centroid of the ring *A* (C1–C6).

*D*—H⋯*A*	*D*—H	H⋯*A*	*D*⋯*A*	*D*—H⋯*A*
C3—H3⋯O1^ix^	0.96 (3)	2.51 (3)	3.268 (2)	136 (2)
C5—H5⋯Cl1^i^	0.96 (2)	2.86 (2)	3.634 (2)	138.8 (17)
C15—H15⋯O1^vi^	0.96 (3)	2.36 (3)	3.270 (2)	159 (2)
C17—H17*A*⋯*Cg*1^i^	0.98 (2)	2.90 (2)	3.619 (2)	131.2 (17)

Symmetry codes: (i) 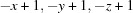; (vi) 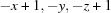; (ix) 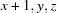.

**Table 2:** Selected interatomic distances (Å)

Cl1⋯C5^i^	3.634 (2)	C21⋯H24*B*	2.86
Cl1⋯C12^ii^	3.548 (2)	C24⋯H21*B*	2.91
Cl1⋯H9	2.82 (3)	C24*A*⋯H24*E* ^viii^	2.44
Cl1⋯H5^i^	2.86 (2)	C24*A*⋯H24*F* ^viii^	2.70
Cl1⋯H12^ii^	2.92 (3)	C24*A*⋯H24*D* ^viii^	1.94
Cl2⋯H20*A* ^iii^	3.13 (2)	H3⋯H17*A* ^ix^	2.42 (4)
Cl2⋯H24*C* ^i^	3.01	H5⋯H17*B*	2.21 (4)
S1⋯N1	3.0439 (16)	H5⋯H16*B*	2.33 (3)
S1⋯C15	3.236 (2)	H12⋯H22*A* ^iii^	2.37
S1⋯H15	2.84 (3)	H16*A*⋯H18*A*	2.47 (3)
S1⋯H2^iv^	3.15 (3)	H16*B*⋯H24*D* ^vii^	2.54
O1⋯C3^v^	3.268 (2)	H16*B*⋯H18*B*	2.46 (3)
O1⋯C17	3.238 (2)	H16*B*⋯H24*A* ^vii^	2.49
O1⋯C15^vi^	3.270 (2)	H17*A*⋯H19*A*	2.59 (3)
O1⋯H3^v^	2.51 (3)	H17*B*⋯H19*B*	2.55 (4)
O1⋯H16*A*	2.43 (2)	H18*B*⋯H20*B*	2.55 (3)
O1⋯H17*A*	2.75 (2)	H19*A*⋯H21*A*	2.58 (4)
O1⋯H9	2.49 (3)	H19*B*⋯H21*B*	2.51 (4)
O1⋯H15^vi^	2.36 (3)	H20*A*⋯H22*A*	2.49
C5⋯C17	3.430 (3)	H20*B*⋯H22*B*	2.54
C5⋯C24^vii^	3.58	H21*A*⋯H23*B*	2.55
C6⋯C24^vii^	3.58	H21*A*⋯H23*C*	2.60
C24*A*⋯C24*A* ^viii^	2.48	H21*B*⋯H24*B*	2.32
C2⋯H19*A* ^i^	2.98 (2)	H21*B*⋯H23*D*	2.34
C5⋯H24*A* ^vii^	2.99	H22*B*⋯H24*C*	2.27
C5⋯H16*B*	2.64 (2)	H22*B*⋯H24*E*	2.43
C5⋯H17*B*	2.93 (3)	H24*D*⋯C24*A* ^viii^	1.94
C7⋯H15^vi^	2.95 (3)	H24*D*⋯H24*D* ^viii^	1.82
C7⋯H17*A*	2.99 (2)	H24*D*⋯H24*E* ^viii^	1.70
C16⋯H5	2.62 (3)	H24*D*⋯H24*F* ^viii^	2.07
C17⋯H3^v^	2.98 (3)	H24*E*⋯H24*F* ^viii^	2.54
C17⋯H5	2.82 (3)		

Symmetry codes: (i) 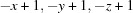; (ii) 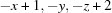; (iii) 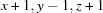; (iv) 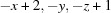; (v) 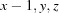; (vi) 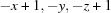; (vii) 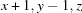; (viii) 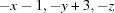; (ix) 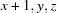.

**Table 3:** Comparison of the selected (X-ray and DFT) geometric data (Å, °)

Bonds/angles	X-ray	B3LYP/6–311G(d,p)
Cl1—C11	1.744 (2)	1.826
Cl2—C13	1.733 (2)	1.821
S1—C8	1.7578 (18)	1.831
S1—C1	1.7589 (18)	1.830
O1—C7	1.228 (2)	1.256
N1—C7	1.368 (2)	1.392
N1—C6	1.420 (2)	1.423
N1—C16	1.479 (2)	1.489
		
C8—S1—C1	97.27 (8)	99.15
C7—N1—C6	123.67 (14)	124.78
C7—N1—C16	117.19 (14)	114.70
C6—N1—C16	119.07 (15)	119.29
C2—C1—C6	120.22 (17)	121.21
C2—C1—S1	119.25 (13)	117.28
C6—C1—S1	120.52 (13)	121.48
C3—C2—S1	120.53 (17)	120.47

**Table 4:** Calculated energies

Mol­ecular Energy (a.u.) (eV)	Compound (I)
Total Energy, *TE* (eV)	−64734
*E*_HOMO_ (eV)	−6.9440
*E*_LUMO_ (eV)	−0.6941
Energy gap, *ΔE* (eV)	6.2499
Dipole moment, *μ* (Debye)	4.4939
Ionization potential, *I* (eV)	6.9440
Electron affinity, *A*	0.6941
Electro negativity, *χ*	3.8191
Hardness, *η*	3.1249
Electrophilicity index, *ω*	2.3337
Softness, *σ*	0.3200
Fraction of electron transferred, *ΔN*	0.5090

**Table 5:** Experimental details

Crystal data	
Chemical formula	C_24_H_27_Cl_2_NOS
*M*_r_	448.42
Crystal system, space group	Triclinic, *P* 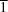
Temperature (K)	150
*a*, *b*, *c* (Å)	8.9961 (3), 10.3755 (3), 13.2565 (4)
α, β, γ (°)	73.857 (1), 88.119 (1), 74.182 (1)
*V* (Å^3^)	1142.32 (6)
*Z*	2
Radiation type	Cu *K*α
μ (mm^−1^)	3.52
Crystal size (mm)	0.20 × 0.14 × 0.08

Data collection	
Diffractometer	Bruker D8 VENTURE PHOTON 100 CMOS
Absorption correction	Multi-scan (*SADABS*; Krause *et al.*, 2015 ▸)
*T*_min_, *T*_max_	0.54, 0.76
No. of measured, independent and observed [*I* > 2σ(*I*)] reflections	8788, 4246, 3772
*R*_int_	0.025
(sin θ/λ)_max_ (Å^−1^)	0.618

Refinement	
*R*[*F* ^2^ > 2σ(*F* ^2^)], *wR*(*F* ^2^), *S*	0.041, 0.107, 1.02
No. of reflections	4246
No. of parameters	349
No. of restraints	14
H-atom treatment	H atoms treated by a mixture of independent and constrained refinement
Δρ_max_, Δρ_min_ (e Å^−3^)	0.32, −0.50

Computer programs: *APEX3* and *SAINT* (Bruker, 2016 ▸), *SHELXT* (Sheldrick, 2015*a* ▸), *SHELXL2018/1* (Sheldrick, 2015*b* ▸), *DIAMOND* (Brandenburg & Putz, 2012 ▸) and *SHELXTL* (Sheldrick, 2008 ▸).

## supplementary crystallographic information

### Crystal data

**Table:**

C_24_H_27_Cl_2_NOS	*Z* = 2
*M**_r_* = 448.42	*F*(000) = 472
Triclinic, *P*1	*D*_x_ = 1.304 Mg m^−^^3^
*a* = 8.9961 (3) Å	Cu *K*α radiation, λ = 1.54178 Å
*b* = 10.3755 (3) Å	Cell parameters from 7317 reflections
*c* = 13.2565 (4) Å	θ = 3.5–72.4°
α = 73.857 (1)°	µ = 3.52 mm^−^^1^
β = 88.119 (1)°	*T* = 150 K
γ = 74.182 (1)°	Column, colourless
*V* = 1142.32 (6) Å^3^	0.20 × 0.14 × 0.08 mm

### Data collection

**Table:**

Bruker D8 VENTURE PHOTON 100 CMOS diffractometer	4246 independent reflections
Radiation source: INCOATEC IµS micro-focus source	3772 reflections with *I* > 2σ(*I*)
Mirror monochromator	*R*_int_ = 0.025
Detector resolution: 10.4167 pixels mm^-1^	θ_max_ = 72.4°, θ_min_ = 3.5°
ω scans	*h* = −10→11
Absorption correction: multi-scan (*SADABS*; Krause *et al.*, 2015)	*k* = −12→12
*T*_min_ = 0.54, *T*_max_ = 0.76	*l* = −13→15
8788 measured reflections	

### Refinement

**Table:**

Refinement on *F*^2^	Primary atom site location: dual space
Least-squares matrix: full	Secondary atom site location: difference Fourier map
*R*[*F*^2^ > 2σ(*F*^2^)] = 0.041	Hydrogen site location: mixed
*wR*(*F*^2^) = 0.107	H atoms treated by a mixture of independent and constrained refinement
*S* = 1.01	*w* = 1/[σ^2^(*F*_o_^2^) + (0.0512*P*)^2^ + 0.6585*P*] where *P* = (*F*_o_^2^ + 2*F*_c_^2^)/3
4246 reflections	(Δ/σ)_max_ < 0.001
349 parameters	Δρ_max_ = 0.32 e Å^−^^3^
14 restraints	Δρ_min_ = −0.50 e Å^−^^3^

### Special details

**Table:**

Geometry. All esds (except the esd in the dihedral angle between two l.s. planes) are estimated using the full covariance matrix. The cell esds are taken into account individually in the estimation of esds in distances, angles and torsion angles; correlations between esds in cell parameters are only used when they are defined by crystal symmetry. An approximate (isotropic) treatment of cell esds is used for estimating esds involving l.s. planes.
Refinement. Refinement of F^2^ against ALL reflections. The weighted R-factor wR and goodness of fit S are based on F^2^, conventional R-factors R are based on F, with F set to zero for negative F^2^. The threshold expression of F^2^ > 2sigma(F^2^) is used only for calculating R-factors(gt) etc. and is not relevant to the choice of reflections for refinement. R-factors based on F^2^ are statistically about twice as large as those based on F, and R- factors based on ALL data will be even larger. The two carbons at the end of the nonyl chain, C23 and C24, are disordered in a 0.562 (4)/0.438 (4) ratio. These were refined with restraints that the two components have comparable geometries. The H-atoms on these carbons as well as those on C22 were included as riding contributions in idealized positions.

### Fractional atomic coordinates and isotropic or equivalent isotropic displacement parameters (Å^2^)

**Table:**

	*x*	*y*	*z*	*U*_iso_*/*U*_eq_	Occ. (<1)
Cl1	0.49354 (7)	0.17182 (5)	0.85050 (4)	0.04871 (16)	
Cl2	0.90576 (10)	−0.32166 (6)	1.00981 (5)	0.0707 (2)	
S1	0.74621 (5)	0.09525 (4)	0.49738 (4)	0.03192 (13)	
O1	0.30841 (14)	0.26156 (14)	0.47052 (11)	0.0350 (3)	
N1	0.49478 (16)	0.35999 (15)	0.39383 (12)	0.0290 (3)	
C1	0.77339 (19)	0.26057 (18)	0.43712 (14)	0.0278 (4)	
C2	0.9224 (2)	0.2768 (2)	0.43323 (15)	0.0330 (4)	
H2	1.005 (3)	0.196 (3)	0.4656 (18)	0.042 (6)*	
C3	0.9469 (2)	0.4057 (2)	0.38810 (17)	0.0380 (4)	
H3	1.051 (3)	0.414 (3)	0.387 (2)	0.052 (7)*	
C4	0.8221 (2)	0.5199 (2)	0.34844 (18)	0.0410 (5)	
H4	0.837 (3)	0.613 (3)	0.316 (2)	0.056 (7)*	
C5	0.6730 (2)	0.5053 (2)	0.35211 (17)	0.0366 (4)	
H5	0.589 (3)	0.587 (2)	0.3270 (18)	0.039 (6)*	
C6	0.64689 (19)	0.37512 (19)	0.39486 (14)	0.0287 (4)	
C7	0.4449 (2)	0.26179 (18)	0.46805 (14)	0.0286 (4)	
C8	0.5633 (2)	0.15326 (18)	0.54591 (15)	0.0291 (4)	
C9	0.5205 (2)	0.1027 (2)	0.64295 (16)	0.0344 (4)	
H9	0.408 (3)	0.145 (3)	0.656 (2)	0.057 (7)*	
C10	0.6193 (2)	−0.0020 (2)	0.73085 (15)	0.0331 (4)	
C11	0.6141 (2)	0.0188 (2)	0.83067 (16)	0.0361 (4)	
C12	0.7034 (3)	−0.0761 (2)	0.91616 (17)	0.0418 (5)	
H12	0.700 (3)	−0.059 (3)	0.986 (2)	0.056 (7)*	
C13	0.8000 (3)	−0.1984 (2)	0.90162 (17)	0.0429 (5)	
C14	0.8106 (3)	−0.2233 (2)	0.80462 (17)	0.0407 (5)	
H14	0.880 (3)	−0.309 (3)	0.797 (2)	0.050 (7)*	
C15	0.7214 (2)	−0.1252 (2)	0.72000 (16)	0.0363 (4)	
H15	0.729 (3)	−0.149 (3)	0.655 (2)	0.046 (6)*	
C16	0.3799 (2)	0.4592 (2)	0.31152 (15)	0.0309 (4)	
H16A	0.315 (3)	0.405 (2)	0.2923 (17)	0.036 (6)*	
H16B	0.434 (2)	0.491 (2)	0.2491 (17)	0.028 (5)*	
C17	0.2769 (2)	0.5837 (2)	0.34303 (15)	0.0313 (4)	
H17A	0.225 (3)	0.548 (2)	0.4065 (18)	0.036 (6)*	
H17B	0.342 (3)	0.636 (2)	0.3630 (17)	0.032 (5)*	
C18	0.1618 (2)	0.6778 (2)	0.25291 (16)	0.0325 (4)	
H18A	0.104 (3)	0.623 (2)	0.2318 (17)	0.036 (6)*	
H18B	0.217 (3)	0.703 (2)	0.1916 (19)	0.040 (6)*	
C19	0.0581 (2)	0.8062 (2)	0.27852 (17)	0.0348 (4)	
H19A	0.004 (3)	0.776 (2)	0.3390 (18)	0.035 (6)*	
H19B	0.123 (3)	0.859 (3)	0.2959 (19)	0.045 (6)*	
C20	−0.0531 (2)	0.9040 (2)	0.18839 (17)	0.0372 (4)	
H20A	−0.125 (3)	0.853 (2)	0.1714 (18)	0.039 (6)*	
H20B	0.010 (3)	0.934 (2)	0.1228 (19)	0.043 (6)*	
C21	−0.1479 (2)	1.0373 (2)	0.21183 (18)	0.0383 (4)	
H21A	−0.205 (3)	1.011 (3)	0.274 (2)	0.059 (8)*	
H21B	−0.080 (3)	1.087 (2)	0.2266 (18)	0.042 (6)*	
C22	−0.2613 (3)	1.1333 (2)	0.12285 (19)	0.0493 (6)	
H22A	−0.325994	1.078902	0.104683	0.059*	
H22B	−0.201418	1.161858	0.060625	0.059*	
C23	−0.3697 (8)	1.2661 (4)	0.1422 (6)	0.0440 (18)	0.562 (4)
H23A	−0.458686	1.305117	0.090467	0.053*	0.562 (4)
H23B	−0.409306	1.245547	0.213838	0.053*	0.562 (4)
C24	−0.2717 (6)	1.3686 (5)	0.1297 (4)	0.0630 (10)	0.562 (4)
H24A	−0.335043	1.455992	0.141231	0.094*	0.562 (4)
H24B	−0.184022	1.328099	0.181244	0.094*	0.562 (4)
H24C	−0.233125	1.387336	0.058565	0.094*	0.562 (4)
C23A	−0.3282 (12)	1.2751 (6)	0.1453 (7)	0.0440 (18)	0.438 (4)
H23C	−0.388048	1.262316	0.209642	0.053*	0.438 (4)
H23D	−0.242430	1.312260	0.158121	0.053*	0.438 (4)
C24A	−0.4333 (8)	1.3801 (6)	0.0534 (5)	0.0630 (10)	0.438 (4)
H24D	−0.474134	1.469164	0.069938	0.094*	0.438 (4)
H24E	−0.373809	1.394060	−0.010101	0.094*	0.438 (4)
H24F	−0.519298	1.344161	0.041374	0.094*	0.438 (4)

### Atomic displacement parameters (Å^2^)

**Table:**

	*U*^11^	*U*^22^	*U*^33^	*U*^12^	*U*^13^	*U*^23^
Cl1	0.0592 (3)	0.0364 (3)	0.0409 (3)	−0.0014 (2)	0.0109 (2)	−0.0080 (2)
Cl2	0.1011 (6)	0.0441 (3)	0.0459 (3)	0.0100 (3)	−0.0229 (3)	−0.0052 (2)
S1	0.0281 (2)	0.0251 (2)	0.0361 (3)	−0.00014 (16)	0.00197 (17)	−0.00517 (17)
O1	0.0233 (6)	0.0393 (7)	0.0427 (8)	−0.0097 (5)	−0.0023 (5)	−0.0104 (6)
N1	0.0200 (7)	0.0281 (7)	0.0351 (8)	−0.0032 (6)	−0.0043 (6)	−0.0054 (6)
C1	0.0231 (8)	0.0284 (8)	0.0289 (9)	−0.0028 (7)	0.0005 (6)	−0.0074 (7)
C2	0.0207 (8)	0.0363 (10)	0.0357 (10)	0.0002 (7)	−0.0005 (7)	−0.0076 (8)
C3	0.0217 (9)	0.0421 (11)	0.0473 (12)	−0.0077 (8)	0.0014 (8)	−0.0088 (9)
C4	0.0275 (9)	0.0339 (10)	0.0569 (13)	−0.0089 (8)	0.0019 (8)	−0.0049 (9)
C5	0.0244 (9)	0.0276 (9)	0.0502 (12)	−0.0032 (7)	−0.0027 (8)	−0.0020 (8)
C6	0.0198 (8)	0.0299 (9)	0.0338 (9)	−0.0038 (7)	−0.0009 (7)	−0.0076 (7)
C7	0.0248 (8)	0.0282 (8)	0.0336 (9)	−0.0058 (7)	−0.0016 (7)	−0.0111 (7)
C8	0.0248 (8)	0.0264 (8)	0.0359 (10)	−0.0063 (7)	−0.0018 (7)	−0.0087 (7)
C9	0.0295 (9)	0.0343 (10)	0.0384 (10)	−0.0093 (8)	0.0010 (8)	−0.0081 (8)
C10	0.0312 (9)	0.0335 (9)	0.0345 (10)	−0.0129 (8)	0.0021 (7)	−0.0055 (8)
C11	0.0403 (10)	0.0294 (9)	0.0358 (10)	−0.0091 (8)	0.0067 (8)	−0.0057 (8)
C12	0.0550 (13)	0.0372 (11)	0.0312 (11)	−0.0117 (9)	0.0019 (9)	−0.0077 (8)
C13	0.0527 (12)	0.0326 (10)	0.0382 (11)	−0.0078 (9)	−0.0058 (9)	−0.0045 (8)
C14	0.0463 (12)	0.0320 (10)	0.0434 (12)	−0.0083 (9)	−0.0026 (9)	−0.0117 (8)
C15	0.0402 (11)	0.0361 (10)	0.0361 (10)	−0.0141 (8)	0.0009 (8)	−0.0122 (8)
C16	0.0240 (8)	0.0312 (9)	0.0326 (10)	−0.0018 (7)	−0.0053 (7)	−0.0058 (7)
C17	0.0230 (8)	0.0306 (9)	0.0374 (10)	−0.0033 (7)	−0.0048 (7)	−0.0080 (8)
C18	0.0253 (9)	0.0322 (9)	0.0359 (10)	−0.0033 (7)	−0.0033 (8)	−0.0067 (8)
C19	0.0295 (9)	0.0320 (10)	0.0396 (11)	−0.0032 (8)	−0.0058 (8)	−0.0089 (8)
C20	0.0332 (10)	0.0318 (10)	0.0406 (11)	0.0006 (8)	−0.0052 (8)	−0.0088 (8)
C21	0.0350 (10)	0.0321 (10)	0.0440 (12)	−0.0017 (8)	−0.0041 (9)	−0.0113 (8)
C22	0.0505 (13)	0.0366 (11)	0.0476 (13)	0.0079 (10)	−0.0053 (10)	−0.0095 (9)
C23	0.033 (4)	0.0350 (13)	0.0547 (15)	0.0018 (17)	0.001 (2)	−0.0088 (11)
C24	0.064 (2)	0.0489 (19)	0.061 (2)	0.0064 (17)	−0.0022 (17)	−0.0126 (16)
C23A	0.033 (4)	0.0350 (13)	0.0547 (15)	0.0018 (17)	0.001 (2)	−0.0088 (11)
C24A	0.064 (2)	0.0489 (19)	0.061 (2)	0.0064 (17)	−0.0022 (17)	−0.0126 (16)

### Geometric parameters (Å, º)

**Table:**

Cl1—C11	1.744 (2)	C16—H16B	0.97 (2)
Cl2—C13	1.733 (2)	C17—C18	1.528 (2)
S1—C8	1.7578 (18)	C17—H17A	0.98 (2)
S1—C1	1.7589 (18)	C17—H17B	0.98 (2)
O1—C7	1.228 (2)	C18—C19	1.521 (3)
N1—C7	1.368 (2)	C18—H18A	0.96 (2)
N1—C6	1.420 (2)	C18—H18B	0.95 (2)
N1—C16	1.479 (2)	C19—C20	1.519 (3)
C1—C2	1.393 (3)	C19—H19A	0.95 (2)
C1—C6	1.398 (2)	C19—H19B	0.97 (3)
C2—C3	1.379 (3)	C20—C21	1.522 (3)
C2—H2	0.96 (2)	C20—H20A	1.00 (2)
C3—C4	1.382 (3)	C20—H20B	1.04 (2)
C3—H3	0.96 (3)	C21—C22	1.515 (3)
C4—C5	1.388 (3)	C21—H21A	0.97 (3)
C4—H4	0.99 (3)	C21—H21B	0.96 (3)
C5—C6	1.392 (3)	C22—C23	1.537 (4)
C5—H5	0.96 (2)	C22—C23A	1.537 (4)
C7—C8	1.497 (2)	C22—H22A	0.9900
C8—C9	1.334 (3)	C22—H22B	0.9900
C9—C10	1.471 (3)	C23—C24	1.530 (7)
C9—H9	1.02 (3)	C23—H23A	0.9900
C10—C11	1.396 (3)	C23—H23B	0.9900
C10—C15	1.397 (3)	C24—H24A	0.9800
C11—C12	1.381 (3)	C24—H24B	0.9800
C12—C13	1.388 (3)	C24—H24C	0.9800
C12—H12	0.99 (3)	C23A—C24A	1.530 (8)
C13—C14	1.376 (3)	C23A—H23C	0.9900
C14—C15	1.385 (3)	C23A—H23D	0.9900
C14—H14	0.97 (3)	C24A—H24D	0.9800
C15—H15	0.96 (3)	C24A—H24E	0.9800
C16—C17	1.525 (3)	C24A—H24F	0.9800
C16—H16A	0.99 (2)		
			
Cl1···C5^i^	3.634 (2)	C21···H24B	2.86
Cl1···C12^ii^	3.548 (2)	C24···H21B	2.91
Cl1···H9	2.82 (3)	C24A···H24E^viii^	2.44
Cl1···H5^i^	2.86 (2)	C24A···H24F^viii^	2.70
Cl1···H12^ii^	2.92 (3)	C24A···H24D^viii^	1.94
Cl2···H20A^iii^	3.13 (2)	H3···H17A^ix^	2.42 (4)
Cl2···H24C^i^	3.01	H5···H17B	2.21 (4)
S1···N1	3.0439 (16)	H5···H16B	2.33 (3)
S1···C15	3.236 (2)	H12···H22A^iii^	2.37
S1···H15	2.84 (3)	H16A···H18A	2.47 (3)
S1···H2^iv^	3.15 (3)	H16B···H24D^vii^	2.54
O1···C3^v^	3.268 (2)	H16B···H18B	2.46 (3)
O1···C17	3.238 (2)	H16B···H24A^vii^	2.49
O1···C15^vi^	3.270 (2)	H17A···H19A	2.59 (3)
O1···H3^v^	2.51 (3)	H17B···H19B	2.55 (4)
O1···H16A	2.43 (2)	H18B···H20B	2.55 (3)
O1···H17A	2.75 (2)	H19A···H21A	2.58 (4)
O1···H9	2.49 (3)	H19B···H21B	2.51 (4)
O1···H15^vi^	2.36 (3)	H20A···H22A	2.49
C5···C17	3.430 (3)	H20B···H22B	2.54
C5···C24^vii^	3.58	H21A···H23B	2.55
C6···C24^vii^	3.58	H21A···H23C	2.60
C24A···C24A^viii^	2.48	H21B···H24B	2.32
C2···H19A^i^	2.98 (2)	H21B···H23D	2.34
C5···H24A^vii^	2.99	H22B···H24C	2.27
C5···H16B	2.64 (2)	H22B···H24E	2.43
C5···H17B	2.93 (3)	H24D···C24A^viii^	1.94
C7···H15^vi^	2.95 (3)	H24D···H24D^viii^	1.82
C7···H17A	2.99 (2)	H24D···H24E^viii^	1.70
C16···H5	2.62 (3)	H24D···H24F^viii^	2.07
C17···H3^v^	2.98 (3)	H24E···H24F^viii^	2.54
C17···H5	2.82 (3)		
			
C8—S1—C1	97.27 (8)	C16—C17—H17B	109.2 (13)
C7—N1—C6	123.67 (14)	C18—C17—H17B	110.8 (12)
C7—N1—C16	117.19 (14)	H17A—C17—H17B	106.0 (18)
C6—N1—C16	119.07 (15)	C19—C18—C17	113.07 (16)
C2—C1—C6	120.22 (17)	C19—C18—H18A	112.5 (13)
C2—C1—S1	119.25 (13)	C17—C18—H18A	109.3 (13)
C6—C1—S1	120.52 (13)	C19—C18—H18B	111.2 (14)
C3—C2—C1	120.53 (17)	C17—C18—H18B	108.8 (14)
C3—C2—H2	122.3 (14)	H18A—C18—H18B	101.4 (19)
C1—C2—H2	117.1 (14)	C20—C19—C18	113.91 (17)
C2—C3—C4	119.53 (18)	C20—C19—H19A	110.8 (14)
C2—C3—H3	118.7 (16)	C18—C19—H19A	108.3 (14)
C4—C3—H3	121.7 (16)	C20—C19—H19B	107.2 (14)
C3—C4—C5	120.51 (19)	C18—C19—H19B	108.7 (15)
C3—C4—H4	120.8 (16)	H19A—C19—H19B	107.7 (19)
C5—C4—H4	118.7 (16)	C19—C20—C21	113.55 (18)
C4—C5—C6	120.58 (17)	C19—C20—H20A	108.8 (13)
C4—C5—H5	118.4 (14)	C21—C20—H20A	109.2 (13)
C6—C5—H5	121.0 (14)	C19—C20—H20B	109.1 (13)
C5—C6—C1	118.59 (16)	C21—C20—H20B	107.1 (13)
C5—C6—N1	120.19 (15)	H20A—C20—H20B	109.1 (19)
C1—C6—N1	121.22 (16)	C22—C21—C20	113.53 (18)
O1—C7—N1	121.48 (16)	C22—C21—H21A	108.6 (17)
O1—C7—C8	121.01 (16)	C20—C21—H21A	107.9 (17)
N1—C7—C8	117.51 (15)	C22—C21—H21B	108.5 (14)
C9—C8—C7	118.51 (17)	C20—C21—H21B	109.6 (14)
C9—C8—S1	125.47 (14)	H21A—C21—H21B	109 (2)
C7—C8—S1	115.89 (13)	C21—C22—C23	117.2 (4)
C8—C9—C10	126.86 (18)	C21—C22—C23A	109.3 (4)
C8—C9—H9	114.5 (15)	C21—C22—H22A	108.0
C10—C9—H9	118.6 (15)	C23—C22—H22A	108.0
C11—C10—C15	116.75 (18)	C21—C22—H22B	108.0
C11—C10—C9	120.39 (18)	C23—C22—H22B	108.0
C15—C10—C9	122.85 (18)	H22A—C22—H22B	107.2
C12—C11—C10	122.93 (19)	C24—C23—C22	105.7 (4)
C12—C11—Cl1	117.26 (16)	C24—C23—H23A	110.6
C10—C11—Cl1	119.80 (15)	C22—C23—H23A	110.6
C11—C12—C13	117.9 (2)	C24—C23—H23B	110.6
C11—C12—H12	121.9 (16)	C22—C23—H23B	110.6
C13—C12—H12	120.1 (16)	H23A—C23—H23B	108.7
C14—C13—C12	121.42 (19)	C23—C24—H24A	109.5
C14—C13—Cl2	120.33 (17)	C23—C24—H24B	109.5
C12—C13—Cl2	118.25 (17)	H24A—C24—H24B	109.5
C13—C14—C15	119.3 (2)	C23—C24—H24C	109.5
C13—C14—H14	119.4 (15)	H24A—C24—H24C	109.5
C15—C14—H14	121.3 (15)	H24B—C24—H24C	109.5
C14—C15—C10	121.63 (19)	C24A—C23A—C22	111.3 (6)
C14—C15—H15	116.3 (15)	C24A—C23A—H23C	109.4
C10—C15—H15	121.9 (15)	C22—C23A—H23C	109.4
N1—C16—C17	115.05 (15)	C24A—C23A—H23D	109.4
N1—C16—H16A	106.7 (13)	C22—C23A—H23D	109.4
C17—C16—H16A	109.8 (13)	H23C—C23A—H23D	108.0
N1—C16—H16B	108.8 (12)	C23A—C24A—H24D	109.5
C17—C16—H16B	110.0 (12)	C23A—C24A—H24E	109.5
H16A—C16—H16B	106.0 (18)	H24D—C24A—H24E	109.5
C16—C17—C18	110.52 (16)	C23A—C24A—H24F	109.5
C16—C17—H17A	107.9 (13)	H24D—C24A—H24F	109.5
C18—C17—H17A	112.2 (13)	H24E—C24A—H24F	109.5
			
C8—S1—C1—C2	147.58 (16)	S1—C8—C9—C10	5.5 (3)
C8—S1—C1—C6	−31.51 (17)	C8—C9—C10—C11	133.9 (2)
C6—C1—C2—C3	0.2 (3)	C8—C9—C10—C15	−46.2 (3)
S1—C1—C2—C3	−178.87 (16)	C15—C10—C11—C12	−0.3 (3)
C1—C2—C3—C4	1.3 (3)	C9—C10—C11—C12	179.60 (19)
C2—C3—C4—C5	−1.1 (3)	C15—C10—C11—Cl1	178.39 (14)
C3—C4—C5—C6	−0.6 (4)	C9—C10—C11—Cl1	−1.7 (3)
C4—C5—C6—C1	2.1 (3)	C10—C11—C12—C13	−1.3 (3)
C4—C5—C6—N1	−177.04 (19)	Cl1—C11—C12—C13	179.99 (17)
C2—C1—C6—C5	−1.9 (3)	C11—C12—C13—C14	1.9 (3)
S1—C1—C6—C5	177.16 (15)	C11—C12—C13—Cl2	−177.07 (17)
C2—C1—C6—N1	177.25 (17)	C12—C13—C14—C15	−1.0 (3)
S1—C1—C6—N1	−3.7 (2)	Cl2—C13—C14—C15	178.02 (17)
C7—N1—C6—C5	−150.53 (19)	C13—C14—C15—C10	−0.7 (3)
C16—N1—C6—C5	26.4 (3)	C11—C10—C15—C14	1.3 (3)
C7—N1—C6—C1	30.3 (3)	C9—C10—C15—C14	−178.57 (19)
C16—N1—C6—C1	−152.76 (17)	C7—N1—C16—C17	82.7 (2)
C6—N1—C7—O1	172.26 (17)	C6—N1—C16—C17	−94.4 (2)
C16—N1—C7—O1	−4.7 (3)	N1—C16—C17—C18	−179.23 (15)
C6—N1—C7—C8	−8.3 (3)	C16—C17—C18—C19	−178.51 (17)
C16—N1—C7—C8	174.73 (16)	C17—C18—C19—C20	177.77 (17)
O1—C7—C8—C9	−32.8 (3)	C18—C19—C20—C21	−175.70 (18)
N1—C7—C8—C9	147.71 (18)	C19—C20—C21—C22	−178.7 (2)
O1—C7—C8—S1	143.15 (15)	C20—C21—C22—C23	176.0 (3)
N1—C7—C8—S1	−36.3 (2)	C20—C21—C22—C23A	−169.4 (5)
C1—S1—C8—C9	−133.86 (18)	C21—C22—C23—C24	78.1 (5)
C1—S1—C8—C7	50.47 (14)	C21—C22—C23A—C24A	175.2 (6)
C7—C8—C9—C10	−178.89 (17)		

Symmetry codes: (i) −*x*+1, −*y*+1, −*z*+1; (ii) −*x*+1, −*y*, −*z*+2; (iii) *x*+1, *y*−1, *z*+1; (iv) −*x*+2, −*y*, −*z*+1; (v) *x*−1, *y*, *z*; (vi) −*x*+1, −*y*, −*z*+1; (vii) *x*+1, *y*−1, *z*; (viii) −*x*−1, −*y*+3, −*z*; (ix) *x*+1, *y*, *z*.

### Hydrogen-bond geometry (Å, º)

*Cg*1 is the centroid of the ring *A* (C1–C6).

**Table:**

*D*—H···*A*	*D*—H	H···*A*	*D*···*A*	*D*—H···*A*
C3—H3···O1^ix^	0.96 (3)	2.51 (3)	3.268 (2)	136 (2)
C5—H5···Cl1^i^	0.96 (2)	2.86 (2)	3.634 (2)	138.8 (17)
C15—H15···O1^vi^	0.96 (3)	2.36 (3)	3.270 (2)	159 (2)
C17—H17*A*···*Cg*1^i^	0.98 (2)	2.90 (2)	3.619 (2)	131.2 (17)

Symmetry codes: (i) −*x*+1, −*y*+1, −*z*+1; (vi) −*x*+1, −*y*, −*z*+1; (ix) *x*+1, *y*, *z*.
